# Chromophorylation of a Novel Cyanobacteriochrome GAF Domain from *Spirulina* and Its Response to Copper Ions

**DOI:** 10.4014/jmb.2009.09048

**Published:** 2020-11-14

**Authors:** Su-Dan Jiang, Yi sheng, Xian-Jun Wu, Yong-Li Zhu, Ping-Ping Li

**Affiliations:** 1 College of Biology and the Environment, Nanjing Forestry University, Nanjing 20037, P.R. China; 2 Collaborative Innovation Center of Sustainable Forestry in Southern China of Jiangsu Province, Nanjing Forestry University, Nanjing 10037, P.R. China; 3 National Positioning Observation Station of Hung-tse Lake Wetland Ecosystem in Jiangsu Province, Hongze, Jiangsu 22100, P.R. China

**Keywords:** Cyanobacteriochrome, phycoerythrobilin, fluorescence quantum yield, biosensor, copper ions, fluorescence quenching

## Abstract

Cyanobacteriochromes (CBCRs) are phytochrome-related photoreceptor proteins in cyanobacteria and cover a wide spectral range from ultraviolet to far-red. A single GAF domain that they contain can bind bilin(s) autocatalytically via heterologous recombination and then fluoresce, with potential applications as biomarkers and biosensors. Here, we report that a novel red/green CBCR GAF domain, SPI1085g2 from *Spirulina subsalsa*, covalently binds both phycocyanobilin (PCB) and phycoerythrobilin (PEB). The PCB-binding GAF domain exhibited canonical red/green photoconversion with weak fluorescence emission. However, the PEB-binding GAF domain, SPI1085g2-PEB, exhibited an intense orange fluorescence (λ_abs.max_ = 520 nm, λ_fluor.max_ = 555 nm), with a fluorescence quantum yield close to 1.0. The fluorescence of SPI1085g2-PEB was selectively and instantaneously quenched by copper ions in a concentration-dependent manner and exhibited reversibility upon treatment with the metal chelator EDTA. This study identified a novel PEB-binding cyanobacteriochrome-based fluorescent protein with the highest quantum yield reported to date and suggests its potential as a biosensor for the rapid detection of copper ions.

## Introduction

Cyanobacteriochromes (CBCRs) are distantly phytochrome-related photoreceptor biliproteins that contain several GAF (cGMP-specific phosphodiesterase/adenylate cyclase/FhlA) domains that act as sensory modules in cyanobacteria [1]. A single GAF domain can bind covalently linear tetrapyrroles and serve as a chromophore. Tetrapyrrole-binding proteins have great spectral diversity and can sense a wide range of wavelengths, from the near-ultraviolet to the near-infrared [2-7]. Most CBCRs utilize phycocyanobilin (PCB) tetrapyrrole as a chromophore and show reversible photoconversion, which is triggered by *Z/E* isomerization of the C15-C16 double bond between the C and D rings of the chromophore [2, 5, 8]. Among PCB-binding CBCRs, red/green CBCR GAF domains that reversibly photoconvert between the red light-absorbing (Pr) thermostable form with 15*Z*-PCB and the green light-absorbing (Pg) metastable form with 15*E*-PCB have been well characterized [9-12]. Recently, some red/green CBCR GAF domains have been shown to covalently bind not only PCB but also biliverdin (BV), extending their spectral range towards longer wavelengths [13]. Further, some red/green CBCR GAF domains can even heterogeneously bind phycoerythrobilin (PEB) and, in terms of fluorescence brightness, exhibit a much higher fluorescence quantum yield than the PCB- and/or BV-binding GAF domains [14]. PEB-binding GAF domains, despite their blue-shifted spectrum, are appropriate for use as platforms for fluorescent protein-based biosensors due to their intense fluorescence emission.

Fluorescent proteins show fluorescence quenching by specific metal ions, and can be used in metal biosensing applications [15-17]. The fluorescence intensity of fluorescent proteins may be reduced, depending on the specific transition metal environment, *i.e.*, the concentration of metal ions. Several fluorescent proteins, including green fluorescent proteins (GFPs), *Discosoma* sp. red fluorescent protein (DsRed), and flavin-binding fluorescent proteins (FbFPs), have been investigated for metal-induced fluorescence quenching and have been suggested as optical biosensors for metal quantitation [18-20]. However, few biosensors based on biliproteins such as CBCRs with intense fluorescence emission are available for the detection of metal ions.

In this article, we identified a novel red/green CBCR GAF domain, SPI1085g2 from *Spirulina subsalsa*, that covalently binds not only PCB but also PEB. PEB-binding SPI1085g2 exhibited an intense orange fluorescence with the highest fluorescence quantum yield reported to date. In addition, we confirmed the selective response of SPI1085g2-PEB fluorescence to copper ions in a concentration-dependent manner, indicating the potential of the red/green CBCR GAF domains for development as a biosensor for copper detection.

## Materials and Methods

### Cloning and Plasmid Construction

The SPI1085g2 (encoding the apoprotein SPI1085g2) gene was amplified from *S. subsalsa* FACHB351 genomic DNA by polymerase chain reaction (PCR) using Pfu DNA polymerase (Tiangen Biotech, China). The sequence used to design the primers was obtained from the GenBank nucleotide sequence database (Accession No. WP_017306776). The primers used to amplify the SPI1085g2 gene were as follows: forward primer, 5′-CTA GAG CTC TTC CCT CGA AAT TGA GCA GAT TTT CC-3′; reverse primer, 5′-GTT AAG CTT AGC GGG TTT TGG CTA ATA ATT CGG C-3′. The SPI1085g2 product was digested with the restriction enzymes SacI and HindIII and inserted into the plasmid pETDuet-SPI1085g3-ho1::pcyA [21], instead of SPI1085g3 to obtain the plasmid pETDuet-SPI1085g2-ho1::pcyA. The pebS gene (encoding PebS), flanked by the restriction enzymes BglII and XhoI, was synthesized by GenScript (China) based on the sequence from the GenBank nucleotide sequence database (Accession No. WP017303809). The pebS gene was then inserted into BglII- and XhoI-digested pETDuet-SPI1085g2-ho1-pcyA instead of pcyA, yielding the plasmid pETDuet-SPI1085g2-ho1::pebS. All the primers were synthesized by GenScript. The sequence of the gene encoding SPI1085g2 was verified by DNA sequencing. The obtained nucleotide and amino acid sequences of SPI1085g2 from *S. subsalsa* FACHB351 are shown in Fig. S1.

### Protein Expression and Purification

The constructed plasmids, pETDuet-SPI1085g2-ho1::pcyA and pETDuet-SPI1085g2-ho1::pebS, were separately transformed into *Escherichia coli* BL21(DE3). The transformed cells were cultured at 37°C in 100 ml Luria-Bertani (LB) medium supplemented with ampicillin (20 μg·ml^−1^). The cells were kept in an ice bath for 30 min after the OD_600_ reached 0.5. After induction with isopropyl β-D-thiogalactoside (1 mM) for 12 h at 18°C, the cells were centrifuged at 12,000 ×*g* for 5 min at 4°C. The cell pellet was resuspended in 5 ml of ice-cold potassium phosphate buffer (KPB, 20 mM, pH 7.2) containing 0.5 M NaCl and disrupted by sonication for 5 min at 200 W (JY92-IIN, Scientz Biotechnology, China). The suspension was centrifuged at 12,000 ×*g* for 15 min at 4°C, and the supernatant was purified via Ni^2+^-affinity chromatography on chelating Sepharose (Amersham Biosciences, Sweden) equilibrated with KPB-containing 0.5 M NaCl. The bound proteins remaining on the column were eluted with 1 ml of saline KPB containing 0.5 M imidazole. After collection, the protein sample was dialyzed twice against saline KPB [22].

### SDS-PAGE and Zn-Induced Fluorescence Assay

Protein samples (50 μl each) were analyzed by polyacrylamide gel electrophoresis (PAGE) in the presence of SDS. The SDS-PAGE gel was composed of a 10% resolving gel and a 5% stacking gel. The samples were boiled with 2×SDS sample buffer containing 30 mM β-mercaptoethanol for 5 min. The purified proteins were visualized by staining with Coomassie blue, and the bilins in the samples were detected by Zn^2+^-induced fluorescence. Fluorescence was visualized through a 630-nm filter upon excitation at 530 nm (GenoSens1850, China).

### Spectral Analysis

All experiments were conducted at room temperature. The absorbance spectra were obtained on a Perkin Elmer Lambda 365 spectrophotometer. After denaturing the proteins in 8 M urea at pH 2.0 in the dark, extinction coefficients were determined by the absorption at: 662 nm (ε = 35,500 M^−1^ cm^−1^) for PCB; 550 nm (ε = 42,800 M^−1^ cm^−1^) for PEB [23]. Red light was provided by a cold fiber optic light source with a 150 W halogen lamp (Bocheng, Nanjing, China) through a bandpass filter with a peak wavelength of 653 nm and a 20-nm half-bandwidth (Rayan, China), while green light was provided by a light source with a peak wavelength of 535 nm and a 40-nm half-bandwidth (Rayan). The light intensity at the sample plane for photoconversion was 15 μmol m^−2^ s^−1^. Samples were irradiated for 3 min for photoconversion. Fluorescence spectra were recorded with a model LS 55 spectrofluorometer (Perkin Elmer, USA). The fluorescence quantum yields were determined in KPB (pH 7.2) using the known ΦF = 0.98 of the biosynthetically obtained CpcB(C-84)-PEB as standards [23]. The biliprotein concentrations were calculated using the Beer-Lambert Law (A = εlc).

### Response Analysis to Heavy Metal

The effect of heavy metal ions on the fluorescence emission of SPI1085g2-PEB was determined by adding various metal ions (CuSO_4_, NiCl_2_, MnCl_2_, ZnSO_4_, CrCl_3_, C°Cl_2_, PbCl_2_ ,CdCl_2_) at a final concentration of 40 μM to 0.25 μM biliprotein in KPB-containing 0.5 M NaCl. After 30 min of incubation at 25°C, the samples were excited at 500 nm, and the emitted fluorescence was measured at 555 nm on a Perkin Elmer LS-55 spectrofluorometer. The fluorescence intensities were measured at 30 sec intervals for 30 min to investigate the response time.

To further investigate the effect of Cu^2+^ on the fluorescence of SPI1085g2-PEB, 10 μl of a Cu^2+^ solution at varying concentrations (0~5 mM) was added to 490 μl of 0.25 μM biliprotein (in KPB-containing 0.5 M NaCl, pH 7.2). After 30 min of incubation at 25°C, the fluorescence at 555 nm of the samples and the emission spectra were recorded after excitation at 500 nm. To assess the reversibility of the fluorescence quenching of SPI1085g2-PEB, simultaneous addition of different concentrations of EDTA to the biliprotein solution with the addition of Cu^2+^ was carried out. After 30 min of incubation at 25°C, the fluorescence emission spectra of the samples were recorded. All experiments were performed in triplicate at 25°C.

### Stern-Volmer Plot and Dissociation Constant

A Stern-Volmer plot was generated for 0.25 μM biliprotein with different concentrations of Cu^2+^ using the equation as follows:

$$F/F_{0}=1+K_{\mathrm{SV}}[Q]$$

Here, *F* is the measured fluorescence intensity in presence of Cu^2+^, *F_0_* is the measured fluorescence intensity without Cu^2+^, [Q] is the concentration of Cu^2+^, and *K*_SV_ is the Stern-Volmer constant. The curve for *F/F_0_* against copper concentration was linearly fitted using the equation, and the slope was determined to give the value of *K*_sv_.

The dissociation constant for copper (*K*_d_) was calculated by plotting the [*ΔF/ΔF*_max_] against the different concentrations of Cu^2+^, fitted using the equation as follows [18]:

$$\Delta F/{\mathrm{\Delta F}}_{\max}=(K_{d}+[P]+[\mathrm{Cu}]-\sqrt{{\left(K_{d}+[P]+[\mathrm{Cu}]-\right)}^{2}-4[P][\mathrm{Cu}]})/2[P],$$

where *ΔF* is the change in measured fluorescence, *ΔF*_max_ is the maximum fluorescence change, *K*_d_ is the dissociation constant, [P] is the protein concentration, and [Cu] is the concentration of Cu^2+^. The curve for *ΔF*/*ΔF*_max_ against copper concentration was fitted using the equation and *K*_d_ was calculated. The strength of interactions between protein and a ligand is inversely related to the value of *K*_d_.

## Results

### Chromophorylation of SPI1085g2

**Fig. 1:**
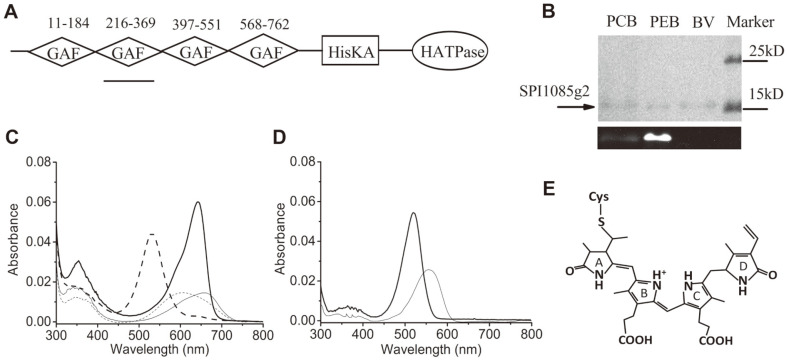
Domain position and chromophorylation of SPI1085g2. (**A**) Domain architecture of SPI9445_RS0121085 (SPI1085). GAF: cGMP-specific phosphodiesterase/adenylate cyclase/FhlA; HisKA: histidine kinase acceptor; HATPase: histidine associated ATPase. The underline indicates the SPI1085g2 position. (**B**) SDS-PAGE analyses of SPI1085g2 chromophorylated with PCB (phycocyanobilin), PEB (phycoerythrobilin) and BV (biliverdin): Coomassie brilliant blue staining (upper gel image) and zinc-induced fluorescence (lower gel image). (**C**) Absorbance spectra of native (heavy lines) and acid-urea-denatured (thin lines) SPI1085g2-PCB. The dashed lines correspond to the 15E state obtained after irradiation with 653/20-nm light, and the solid lines correspond to the 15Z state obtained after irradiation with 530/20-nm light. (**D**) Absorbance spectra of native (heavy line) and acid-urea-denatured (thin line) SPI1085g2-PEB. (**E**) The structure of phycoerythrobilin (PEB).

SPI1085, a hypothetical signal transduction protein from *S. subsalsa*, contains four GAF domains, as previously reported, one of which, SPI1085g3, can bind PCB as a chromophore and exhibits a red/dark fluorescence switch [21]. Here, we cloned the DNA segment encoding the second GAF domain of SPI1085 (SPI1085g2, residues 216-369) from the *S. subsalsa* FACHB351 genome (Fig. 1A). To evaluate the bilin-binding capability of SPI1085g2, we first tested the chromophorylation of SPI1085g2 with PCB. SPI1085g2 can covalently bind PCB, as demonstrated by the zinc-dependent fluorescence observed by SDS-PAGE (Fig. 1B), despite exhibiting a low bilin-binding rate, and by spectral and acid denaturation analysis of chromophorylated SPI1085g2 (Fig. 1C). The PCB-binding SPI1085g2 (SPI1085g2-PCB) showed reversible photoconversion between a red-absorbing Pr form at 642 nm and a green-absorbing Pg form at 535 nm (Fig. 1C), which was very similar to that of Slr1393g3 from *Synechocystis* sp. PCC 6803 [10]. We compared the primary sequence of SPI1085g2 with those of Slr1393g3 and another red/green CBCR GAF domain, All2699g3 [24]. Some residues conserved or enriched in typical red/green CBCR GAF domains appear in SPI1085g2 (Fig. S2). Therefore, SPI1085g2 belongs to the canonical red/green CBCRs. Next, chromophorylation with PEB instead of PCB as the chromophore was performed and unexpectedly resulted in highly efficient binding of PEB to SPI1085g2, as indicated by a band with strong zinc-dependent fluorescence (Fig. 1B) and spectral and acid denaturation analysis (Fig. 1D). Furthermore, we failed to obtain chromophorylated SPI1085g2 with BV as demonstrated by the zinc-dependent fluorescence (Fig. 1B). The PEB-binding SPI1085g2 (SPI1085g2-PEB) was not photochromic and exhibited maximum absorption at 520 nm (Fig. 1D). Although PEB also covalently binds to Slr1393g3 and All2699g3, they are partially converted into phycourobilin (PUB), which results in a lower yield [14]. The PEB bound to SPI1085g2 was not further modified. Therefore, SPI1085g2-PEB was more similar to the previously reported PEB-binding All2699g1 (All2699g1-PEB) but with a remarkably blue-shifted absorption maximum compared to All2699g1 with absorption maximum at 575 nm [14]. Clearly, the PEB-binding GAF domains could be divided into two categories, Slr1393g3 and All2699g3 with isomerization of PEB to PUB and lower yield, and SPI1085g2 and All2699g1 with non-isomerization and higher yield, respectively. We compared the amino acid sequences of the four GAF domains based on multiple alignment (Fig. S2). SPI1085g2 and All2699g1 showed great differences in some conserved amino acid residues. It was speculated that some identical residues in SPI1085g2 and All2699g1 might play important roles for PEB incorporation.

### Intense Fluorescence of SPI1085g2-PEB

**Fig. 2:**
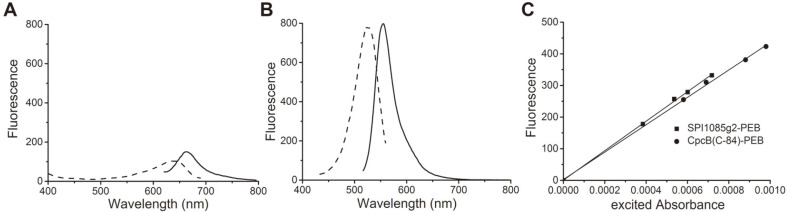
Fluorescence properties of chromophorylated SPI1085g2. Fluorescence excitation (dashed line) and emission (solid line) spectra of SPI1085g2-PCB, excited at 600 nm (**A**), and SPI1085g2-PEB, excited at 480 nm (**B**). (**C**) Plot of fluorescence emission vs. excited absorbance for the dilution series of SPI1085g2-PEB (solid line) and CpcB(C-84)-PEB (dashed line). The slopes of these lines are proportional to the quantum yields for fluorescence.

To study the fluorescence properties of chromophorylated SPI1085g2, fluorescence spectra at room temperature were measured. The results showed that the thermostable forms of SPI1085g2-PCB exhibited only weak fluorescence with an emission maximum at 662 nm (Fig. 2A), whereas SPI1085g2-PEB exhibited an intense orange fluorescence with an emission maximum at 555 nm (Fig. 2B). Furthermore, the fluorescence quantum yield of SPI1085g2-PEB was calculated to be very close to 1.0 (Table 1), which was comparable with that of some phycobiliproteins attached to PEB (Fig. 2C) but higher than that of All2699g1 (ΦF = 0.55) and Cph1 (ΦF = 0.72)[14, 23, 25]. Moreover, SPI1085g2-PEB also has a larger Stokes shift (35 nm) than PEB-binding phycobiliproteins All2699g1 and Cph1 for the reduction of scatter interference.

### Response of SPI1085g2-PEB to Copper

**Fig. 3:**
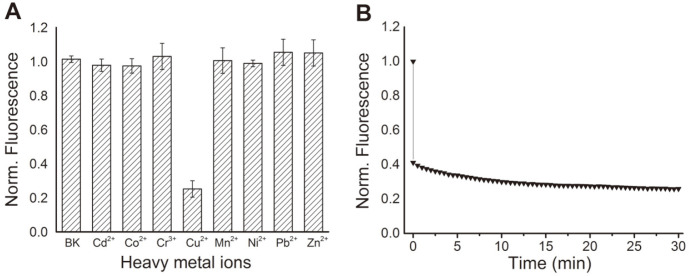
Selectivity and response time of SPI1085g2-PEB. (**A**) Effects of different heavy metal ions on the fluorescence emission of SPI1085g2-PEB. (**B**) Response time of SPI1085g2-PEB in the presence of Cu^2+^.

To evaluate the potential of SPI1085g2 as a single fluorescent protein biosensor for heavy metal detection, we conducted spectroscopic studies to investigate whether SPI1085g2-PEB can be fluorescently quenched by various heavy metal ions. The results demonstrated that the fluorescence of SPI1085g2-PEB was not substantially affected by the addition of 0.04 mM heavy metal ions, such as Cd^2+^, Co^2+^, Cr^3+^, Mn^2+^, Ni^2+^, Pb^2+^, and Zn^2+^. However, the addition of 0.04 mM Cu^2+^ exerted a maximum fluorescence quenching of approximately 75% (Fig. 3A). Therefore, among the tested heavy metal ions, only Cu^2+^ caused selective fluorescence quenching of SPI1085g2-PEB. Once the Cu^2+^ was added into SPI1085g2-PEB, the fluorescence intensity at 555 nm was recorded over time (0-30 min) to investigate the response time of SPI1085g2-PEB toward Cu^2+^. A large instantaneous decrease in fluorescence intensity was observed within less than 5 sec after adding Cu^2+^ and the intensity continued to decline slowly over time (Fig. 3B). This indicates that SPI1085g2-PEB exhibited very short response time to Cu^2+^ and provided an advantage for biosensor applications.

**Fig. 4:**
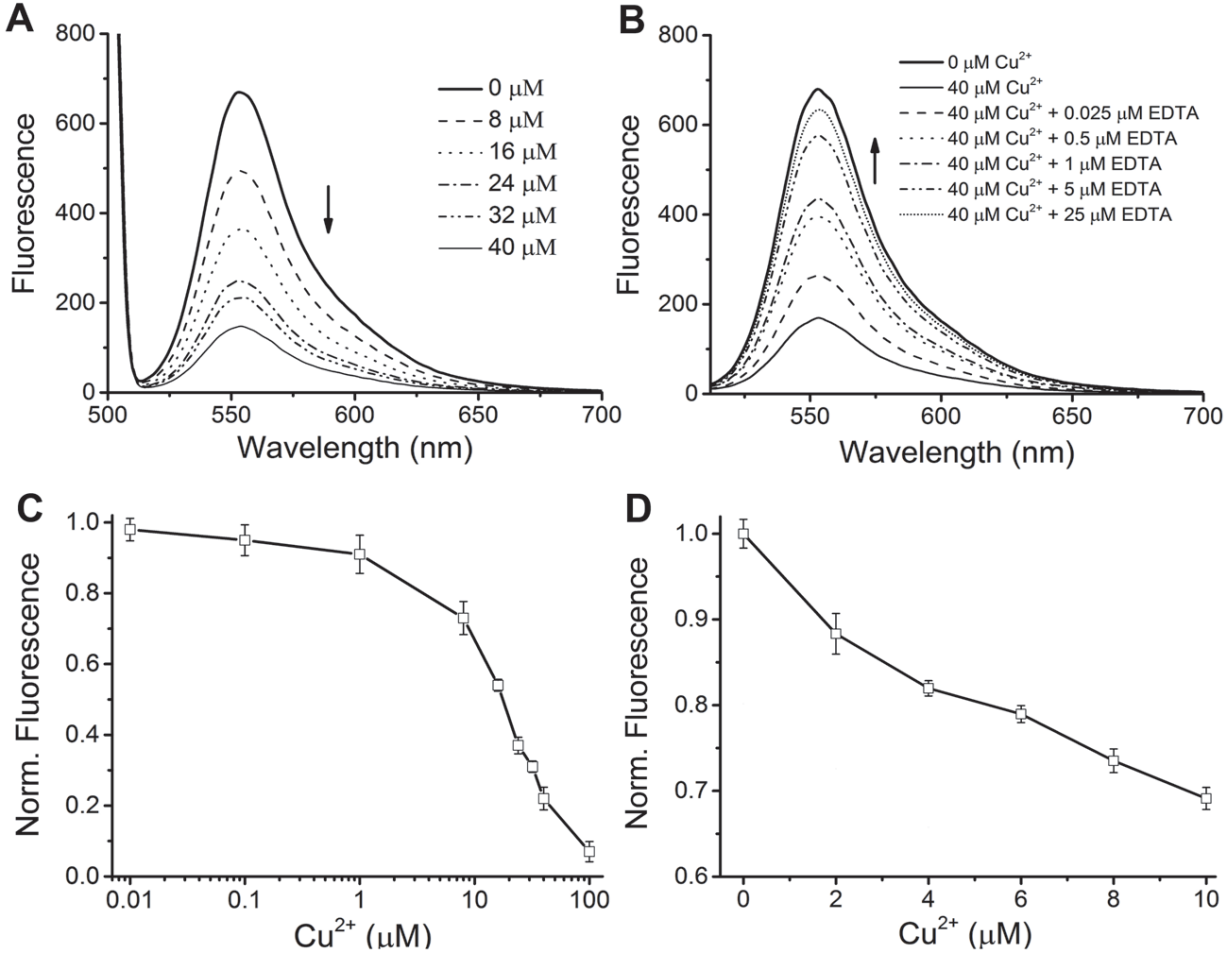
Response of SPI1085g2-PEB in a concentration-dependent manner. (**A**) Effect of the presence of different copper concentrations on the fluorescence emission of SPI1085g2-PEB (0.25 μM). (**B**) Reversibility of the Cu^2+^-mediated quenching of SPI1085g2-PEB. (**C**) Dose-response curve of SPI1085g2-PEB against Cu^2+^ in a broad range from 0.01 to 100 μM. (**D**) Dose-response curve generated for SPI1085g2-PEB with different concentrations of Cu^2+^ in a narrower range from 2 to 10 μM.

Next, the responding properties of SPI1085g2-PEB for Cu^2+^ were analyzed by incubating equimolar amounts of purified fluorescent proteins with 0-40 μM Cu^2+^. The fluorescence emission spectrum of SPI1085g2-PEB indicated a reduction of fluorescence intensity with increasing Cu^2+^ concentration, while the shape of the emission signal and its position did not change (Fig. 4A). To confirm that the fluorescence quenching we observed was reversible, we treated mixtures of 0.25 μM SPI1085g2-PEB and 40 μM Cu^2+^ with 0.025-25 μM EDTA. We found that the fluorescence emission spectrum of the quenched fluorescent proteins gradually reverted close to the initial state with the increase of the EDTA concentration (Fig. 4B), demonstrating that the quenching effect was due to the presence of the metal ion.

A more detailed analysis of fluorescence quenching as a function of the copper concentration for SPI1085g2-PEB was performed over a broad concentration range from 0.01 to 100 μM. The results showed that the fluorescence intensity decreased with increasing copper concentration (Fig. 4C). Only a 10% decrease in emission was observed when 1 μM Cu^2+^ was added, yet Cu^2+^ in the range of 1 to 100 μM caused a remarkable decrease of fluorescence intensity. Furthermore, there was a sensitive dose-effect relationship between the fluorescence intensity and the concentration of Cu^2+^ over a narrower range from 2 to 10 μM, and a decrease of fluorescence intensity with increasing copper concentration was clearly observed (Fig. 4D). The data were fitted to calculate the dissociation constant of SPI1085g2-PEB. The dissociation constant of the copper binding site on the rAsGFP (recombinant *Anemonia sulcata* green fluorescent protein) was identified as 14.18 ± 0.21 μM, which indicates the affinity of SPI1085g2-PEB for Cu^2+^. The copper dissociation constant of SPI1085g2-PEB was similar to that of DsRed mutant drFP583 [26], but higher than that of DsRed, GFPdopa, and CreiLOV [18-20].

**Fig. 5:**
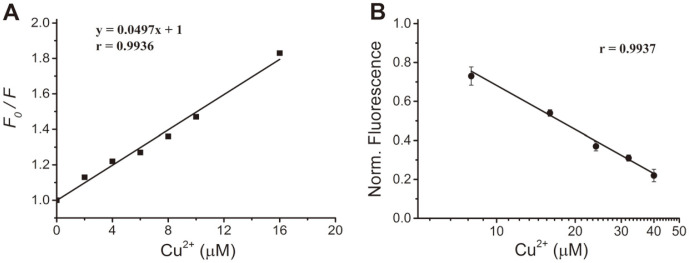
Linear relationship of SPI1085g2-PEB against Cu^2+^ in the micromolar range. (**A**) Stern–Volmer plot generated for SPI1085g2-PEB by the addition of Cu^2+^ at different concentrations. (**B**) The plot of the normalized fluorescence intensity of SPI1085g2-PEB vs. Cu^2+^ concentration (8-40 μM). Measurements were repeated 3 times and data were fitted linearly.

To exhibit a linear dose-effect relationship, a Stern–Volmer plot was generated at room temperature and a linear plot was obtained with a correlation coefficient of 0.9936 (Fig. 5A). The *K*_SV_ value was evaluated as 0.5×10^5^ M^−1^ according to the slope of the fitted straight line.. Meanwhile, the dose response curve of Cu^2+^ exposure (8-40 μM) demonstrated excellent linearity with a correlation coefficient of 0.9937 (Fig. 5B). This sensitivity covers the maximum allowed copper concentration in drinking water suggested by the World Health Organization,(31.15 μM) [27], above which aqueous solution may pose a threat to human health. These observations indicate that SPI1085g2-PEB has potential as a biosensor to be applied to the detection of copper ions.

## Discussion

CBCRs chromophorylated with different tetrapyrrole chromophores exhibit a great diversity of spectral properties [6]. In the current study, we tested the chromophorylation of a novel red/green CBCR GAF domain of *Spirulina*, SPI1085g2. This GAF domain binds PCB but cannot bind BV. PEB chromophorylated SPI1085g2 exhibits a fluorescence quantum yield close to 1.0, higher than those of all reported biliproteins. SPI1085g2-PEB was not photochromic with intense orange fluorescence, similar to the previously reported All2699g1-PEB [14]. However, SPI1085g2-PEB exhibited blue-shifted absorption and emission spectra from 575 to 520 nm in absorption maximum and from 586 to 555 nm in emission maximum compared with All2699g1-PEB. Moreover, SPI1085g2-PEB had a larger Stokes-shift and higher fluorescence quantum yield close to unity than those of All2699g1-PEB. This novel GAF domain expands the spectral variation of biliprotein-based fluorescent constructs and can be used in combination with All2699g1 in flexible applications, such as dual-color labeling and FRET experiments. In terms of both the wavelengths of excitation and emission and fluorescence quantum yield, SPI1085g2-PEB was very similar to CpcB-PEB, a previously reported orange fluorescing phycobiliprotein [23]. However, the chromophorylation of CBCR GAF domains does not require the involvement of a lyase, facilitating chromophore assembly, compared to that of phycobiliproteins. SPI1085g2-PEB, which has the most intense fluorescence quantum yield reported to date, may be a promising developmental platform for fluorescent biosensor devices.

Direct conversion of fluorescent proteins into a sensor tool for detecting metal ions is currently attracting much interest. We investigated the possibility of using SPI1085g2-PEB as a single biosensor to detect heavy metal ions. The fluorescence of SPI1085g2-PEB can be selectively and instantaneously quenched by Cu^2+^ in a concentration-dependent manner and recovered almost to the initial fluorescence with the addition of EDTA. SPI1085g2-PEB provided a much faster response time compared to other fluorescent proteins, such as His6GFP (chimeric green fluorescent protein harboring hexahistidine) [28], rAsGFP [29], and iLOV (variant derived through engineering of the light, oxygen or voltage sensing domain) [30], which suggests the potential of SPI1085g2-PEB as a biosensor for rapid detection of copper ions. In this case, the observed fluorescence changes may be due to the interaction between Cu^2+^ and metal-binding sites, which presumably cause static quenching, as well as that of His6GFP, which bring copper close to the chromophore of the proteins to form a nonfluorescent ground state complex. Consistent with this hypothesis, the emission spectrum of SPI1085g2-PEB did not change in shape or position, but a decrease in intensity was clearly demonstrated. The PEB chromophore unit may be considered as a binding site for Cu^2+^, because previous studies tend to suggest the tetrapyrrole core as the probable binding site for heavy metal ions [31]. On the other hand, the intramolecular histidine and cysteine residues and the polyhistidine-tag at the N-terminus of SPI1085g2-PEB may also be responsible for binding copper since copper ions prefer to coordinate with nitrogen and thiolate groups typically present in the side chains of histidine and cysteine [32]. In brief, copper ions are bound to either the tetrapyrrole chromophore or the amino-acid residues, or both. This remains to be proved.

The fluorescence reversibility of SPI1085g2-PEB suggests that the continuous and reproducible monitoring of exchangeable copper ions is possible with the protein, which is important for cost-effective application of biosensor materials. The fluorescence recovery of Cu^2+^-binding SPI1085g2-PEB in the presence of some specific molecules could extend the applications of SPI1085g2-PEB into the detection of mercapto-biomolecules such as homocysteine [33]. SPI1085g2-PEB can be integrated into some immobilized carriers, such as Sol-gel [27], amine-coated glass surfaces [18], and nanocellulose matrices [34], enhancing its stability and facilitating reutilization of the systems. A very small amount of SPI1085g2-PEB is required for the response to copper due to its higher fluorescence quantum yield than those of GFP derivatives [15, 27], iq-FPs [16], DsRed [19], HeRed [35], Dronpa [36], ZsYellow [37], and CreiLOV [20]. Furthermore, a large number of CBCRs have been identified and may provide a set of available libraries for single fluorescent protein biosensors due to their novel photophysical and biochemical properties.

## Supplemental Materials



Supplementary data for this paper are available on-line only at http://jmb.or.kr.

## Figures and Tables

**Table 1:** Spectral properties of reconstituted biliproteins.

Biliprotein	Absorbance		Fluorescence	

	λ_max_[nm]	εvis [M^-1^·cm^-1^] × 10^-4^	λ_max_ [nm]	ФF
SPI1085g2-PEB	520	7.4	555	0.996
SPI1085g2-PCB	642	8.3	662	0.007

Spectra were obtained in potassium phosphate buffer (20 mM, pH 7.0). Extinction coefficients and fluorescence yields were averages of two independent experiments.
