# Supplementary material for: Chromophorylation of a Novel Cyanobacteriochrome GAF Domain from Spirulina and Its Response to Copper Ions
Source: J Microbiol Biotechnol. 2020 Nov 14;31(2):233–9. doi: 10.4014/jmb.2009.09048 (PMC9705869; doi:10.4014/jmb.2009.09048)
Supplement: Supplementary file 1 [file jmb-31-2-233-supple.pdf]

J. Microbiol. Biotechnol.
https://doi.org/10.4014/jmb.2009.09048

```
646   ATTGAGCAGATTTTCCGCACCAGTACCGAAGAAGTCCGTCAGGTATTACAAGCGGAACGA
216    I  E  Q  I  F  R  T  S  T  E  E  V  R  Q  V  L  Q  A  E  R

706   GTGGCTATTTATCGCTTTTTCCCCGACTGGAGTGGGGAATTTGTGGCGGAATCTAAAGGG
236    V  A  I  Y  R  F  F  P  D  W  S  G  E  F  V  A  E  S  K  G

766   GAGGAGTGGTGTAGTTTAGTGGGGGGAGAACAGCCCATTATTGCCGACACCCACCTTCAG
256    E  E  W  C  S  L  V  G  G  E  Q  P  I  I  A  D  T  H  L  Q

826   GAAACCCAAGGGGGGCGTTATGTTCACGGGGAAACCTTCGCTATTGATGACATCTACTTA
276    E  T  Q  G  G  R  Y  V  H  G  E  T  F  A  I  D  D  I  Y  L

886   GCGGGGCATCAGGACTGTCATATTGCCCTGTTAGAGCAGTTTCAAGCCCGCGCCTATGTG
296    A  G  H  Q  D  C  H  I  A  L  L  E  Q  F  Q  A  R  A  Y  V

946   ATTGTACCCATTTTACATGGAGAGCAGTTGTGGGGCCTTTTAGCGGCCTATCAGAACTCT
316    I  V  P  I  L  H  G  E  Q  L  W  G  L  L  A  A  Y  Q  N  S

1006  GGTCCCCGACGTTGGGAAAGCCACGAGGTGGACTTATTAGCCCAAATTGGGCGACAGTTA
336    G  P  R  R  W  E  S  H  E  V  D  L  L  A  Q  I  G  R  Q  L

1066  GCCATTGCGCTCCAACAAGCCGAATTATTAGCCAAAACCCGC
356    A  I  A  L  Q  Q  A  E  L  L  A  K  T  R
```

Fig. S1. Nucleotide and amino acid sequence of SPI1085g2 (GAF2, 216-369aa) from *S. subsalsa* FACHB351. Boxes show the conserved motifs in CBCR GAF domains.

```
All2699g3(484-537)    DRVSVYRFDNEWGGEFVGD--FEATSPHWSNESKISIN-TVWNDTYLQNTQGGRYRY
Slr1393g3(459-511)    DRVLVYRFNPDWSGEFIH----ESVAQMWEPLKDLQNNFPLWQDTYLQENEGGRYRN
All2699g1 (50-106)    DRVMIYKFHPDGSGQVIAESIHENRLPSLLGLNFPADDIPPQARELLVKSKVRSIVD
SPI1085g2(234-284)    ERVAIYRFFPDWSGEFVA----ESKGEEWCS-LVGGEQ-PIIADTHLQETQGGRYVH

All2699g3(538-593)    NETFAVD-DIYKVGFTQCHVENLEQFQIYAFVLAPIFVGQKLWGLLATYQHSGPRQW
Slr1393g3(512-567)    HESLAVG-DVETAGFTDCHLDNLRRFEIRAFLTVPVFVGEQLWGLLGAYQNGAPRHW
All2699g1(107-176)    VATGMIG*DICYRPVDSCHVEYLTAMGVKSSVVAPIFCQDELWGLLVSHHSEN-RTV
SPI1085g2(285-340)    GETFAID-DIYLAGHQDCHIALLEQFQARAYVIVPILHGEQLWGLLAAYQNSGPRRW
```

Fig. S2. Sequence alignment of SPI1085g2 and reported PEB-binding red/green CBCR GAF domains. Highly conserved residues in red/green CBCR GAF domains, black; CBCR GAF domains with the partially modified PEB, blue; CBCR GAF domains with the unmodified PEB, orange; Residues conserved in the typical red/green CBCR GAF domains, green. The canonical Cys residues that bind bilins are highlighted in red. The residues highlighted in light yellow are presumed to be important for PEB incorporation and high fluorescence quantum yield. The asterisk (*) indicates omitted non-conserved amino acids.
